# In search of an international multidimensional action plan for second victim support: a narrative review

**DOI:** 10.1186/s12913-023-09637-8

**Published:** 2023-07-31

**Authors:** Deborah Seys, Massimiliano Panella, Sophia Russotto, Reinhard Strametz, José Joaquín Mira, Astrid Van Wilder, Lode Godderis, Kris Vanhaecht

**Affiliations:** 1 Department Public Health and Primary Care, KU Leuven, Kapucijnenvoer 35, Leuven, Belgium; 2 Department Public Health and Primary Care, KU Leuven, Leuven, Belgium; 3 Department of Translational Medicine, University of Eastern Piedmont, Novara, Italy; 4 RheinMain University of Applied Science, Wiesbaden, Germany; 5 The Foundation for the Promotion of Health and Biomedical Research of Valencia Region, Alicante, Spain; 6 Health Psychology Department, Miguel Hernandez University, Elche, Spain; 7 External Service for Prevention and Protection at Work, IDEWE, Heverlee, Belgium; 8 Department of Quality, University Hospitals Leuven, 3000 Leuven, Belgium

**Keywords:** Healthcare professionals, Patients safety incident, Second victim, Support, Impact

## Abstract

**Background:**

Insights around second victims (SV) and patient safety has been growing over time. An overview of the available evidence is lacking. This review aims to describe (i) the impact a patient safety incident can have and (ii) how healthcare professionals can be supported in the aftermath of a patient safety incident.

**Methods:**

A literature search in Medline, EMBASE and CINAHL was performed between 1 and 2010 and 26 November 2020 with studies on SV as inclusion criteria. To be included in this review the studies must include healthcare professionals involved in the aftermath of a patient safety incident.

**Results:**

In total 104 studies were included. SVs can suffer from both psychosocial (negative and positive), professional and physical reactions. Support can be provided at five levels. The first level is prevention (on individual and organizational level) referring to measures taken before a patient safety incident happens. The other four levels focus on providing support in the aftermath of a patient safety incident, such as self-care of individuals and/or team, support by peers and triage, structured support by an expert in the field (professional support) and structured clinical support.

**Conclusion:**

The impact of a patient safety incident on healthcare professionals is broad and diverse. Support programs should be organized at five levels, starting with preventive actions followed by self-care, support by peers, structured professional support and clinical support. This multilevel approach can now be translated in different countries, networks and organizations based on their own culture, support history, structure and legal context. Next to this, they should also include the stage of recovery in which the healthcare professional is located in.

**Supplementary Information:**

The online version contains supplementary material available at 10.1186/s12913-023-09637-8.

## Introduction

Since the publication of “To Err is Human” by the Institute of Medicine (IOM) back in 1999, attention to patient safety has been surging [1]. Several actions have been undertaken to reduce the number of preventable errors and patient safety incidents (PSIs) in the hospital. As mentioned in the Global Patient Safety Action Plan 2021–2030 (Strategy 4.4) by the WHO, attention for PSIs continues to be important and it needs to be “ensured that patients, families and health care staff are given ongoing psychological and other support in the aftermath of a serious PSI” [2]. Next to the ‘first victim’, i.e. the patient and their kin affected by the PSI, the health care staff is considered to be ‘the second victim’ (SV), a term introduced by Wu in 2000 [3]. Since Wu’s seminal publication in BMJ, research on the impact of a PSI on the SV and on how to support them in the most optimal way has been numerous with collective consensus that the SV should not be treated punitively [4–8].

The term ‘second victims’ has been in use for over two decades and has been on the receiving end of some criticism [9–12]. Recently, the definition has been further improved upon by the COST Action on European Researchers’ Network Working on Second Victims (ERNST) [13] and a SV is now defined as “Any health care worker, directly or indirectly involved in an unanticipated adverse patient event, unintentional healthcare error, or patient injury, and who becomes victimized in the sense that they are also negatively impacted” [12]. In the aftermath of a PSI, healthcare professionals are highly affected by different psychological and psychosomatic symptoms, e.g. troubling memories, anxiety, anger, remorse, distress and fear of future errors [4, 5, 14].

When a healthcare professional is involved, the symptoms in the aftermath of a PSI can differ dependent on the stage of recovery healthcare professionals find themselves in. Scott et al. described the natural history of recovery in the aftermath of a PSI to first start with a chaos and accident response (1), followed by intrusive reflections (2), restoring personal integrity (3), enduring the inquisition (4), obtaining emotional first aid (5) and finally moving on (6) [15]. The different recovery stages described are comparable with the different stages of grief in the aftermath of a trauma as described by Kübler-Ross, which are denial, anger, bargaining, depression and acceptance [16]. This implies that being involved in a PSI is associated with a spiral of emotions [17]. Finally, a PSI can have a long impact on the healthcare professional which can be negative when symptoms are getting worse or the duration lasts longer than expected [18].

Since 2006, several support resources have been described in the literature, most established in the United States, trying to help healthcare professionals in dealing with the impact of a PSI [19]. In 2013, a review with possible support mechanisms was published concluding that support can be provided on the individual and on organizational level. These SV programmes should include support to be provided immediately after PSI occurrence as well as on the middle long and long term [4]. Scott et al. described the “Scott three-tiered emotional support system”, which includes department support and leadership mentoring (Tier 1), support by peer experts (Tier 2) and professional resources (Tier 3) [20]. However, the recent attention given towards assessments of a ‘just culture’ indicate that a lot of work is still required to put this into practice [21]. A recent meta-analysis confirmed that second victims use different types of coping strategies, e.g. being task-oriented or avoidance-oriented, to deal with the emotional impact of a PSI [8, 22]. Further actions to increase awareness about the second victim after a PSI and implement support protocols are necessary to improve the complex and multi-layered impact of a PSI [23].

As the evidence-base around SVs and patient safety has evolved, and several of the available literature reviews were published over ten years ago, the international community lacks an update of the available insights and findings. How healthcare professionals can reduce the impact of a PSI has not been explored within current evidence-base. Therefore, this review wants to describe the different kinds of impact a PSI can cause, not only on a personal and physical level, but also on a professional level. Additionally, it wants to recount which support and prevention strategies are required. The following two research questions were defined: (i) What kind of impact of PSIs on healthcare professionals can be identified? and (ii) How can healthcare professionals be supported in the aftermath of a PSI?

## Methods

### Information sources and search strategy

A literature search was performed in Medline, EMBASE and CINAHL from 1 October 2010 onwards, following on two reviews published by Seys et al. [4, 5], and collected literature until 26 November 2020. The following search strategy was employed: (“second victim,” OR “medical error” OR “adverse event”) AND (“psychology” OR “emotions” OR “feelings” OR “burnout” OR “depression” OR “empathy” OR “attitude of health personnel,”) OR “medical error”[MeSH] AND “Burnout, Professional”[MeSH] OR “Depressive Disorder”[MeSH] OR “Empathy”[MeSH] for Medline. For EMBASE: (“second victim,” OR “medical error” OR “adverse event”) AND (“psychology” OR “emotions” OR “feelings” OR “burnout” OR “depression” OR “empathy” OR “attitude of health personnel,”) OR “medical error”/exp AND “professional burnout’/exp OR “depression’/exp OR ‘empathy’/exp. For CINAHL: (“second victim,” OR “medical error” OR “adverse event”) AND (“psychology” OR “emotions” OR “feelings” OR “burnout” OR “depression” OR “empathy” OR “attitude of health personnel,”). This study was conducted according to the Preferred Reporting Items for Systematic Reviews and Meta-analyses (PRISMA) standard [24]. The studies should include healthcare professionals involved in a patient safety incident (PSI), where a PSI is defined as “an event or circumstance that could have resulted, or did result, in unnecessary harm to a patient.” [25].

### Eligibility criteria

Studies were included if any of the following criteria were met. (i) They described a definition or described the term second victims. (ii) They mentioned the prevalence of health care professionals involved in a PSI, which could have occurred over the time span of their career or during a well-defined period. (iii) They studied the impact of a PSI on the involved health care professional without restrictions regarding the level of impact or harm. (iv) Finally, studies were included if they assessed what support was provided and/or needed in the aftermath of a PSI.

We excluded studies not published in English, reviews, conference reports, newspaper stories, and anecdotal evidence. Publications on analyses occurring during the COVID-19 pandemic were excluded due to the fact that these publications are no longer able to purely distinguish the effect of a PSI on the second victim, but have COVID-19 as a confounding factor.

### Article screening

All citations were imported into the citations manager, Endnote X9, and duplicates were initially removed by this citation manager. Duplicates found during the title analyses were removed manually. In the next phase, the titles and abstracts were first screened by two reviewers (DS, KV) to eliminate unrelated studies. Any discrepancies were resolved by two other investigators (MP, SR). For all remaining relevant articles, the full text was retrieved, and two reviewers examined them independently according to the eligibility criteria.

### Qualitative analysis: data extraction and data synthesis

Data were extracted from the full text by a single investigator (DS) using a data extraction sheet. The sheet included study summary information (authors, country, year of study), study design, patient population reports (sample size, type of respondents) and outcome measurements (impact of PSIs, support in the aftermath of a PSI). The outcome measurements related to the impact of a PSI were clustered by the researchers into psychosocial reactions, positive professional reactions, negative professional reactions or physical reactions which the authors defined before the study.Each of the outcomes related to support in the aftermath of a PSI were extracted from each article. Based on these results the support in the aftermath of a PSI could be clustered into five separate levels, whereby clustering occurred on the degree of support received by SVs. The data extraction was thoroughly discussed with the members of the review team (DS, KV, SR, MP).

### Patient and public involvement

Patients and/or the public were not involved in the design, or conduct, or reporting, or dissemination plans of this research.

## Results

**Fig. 1:**
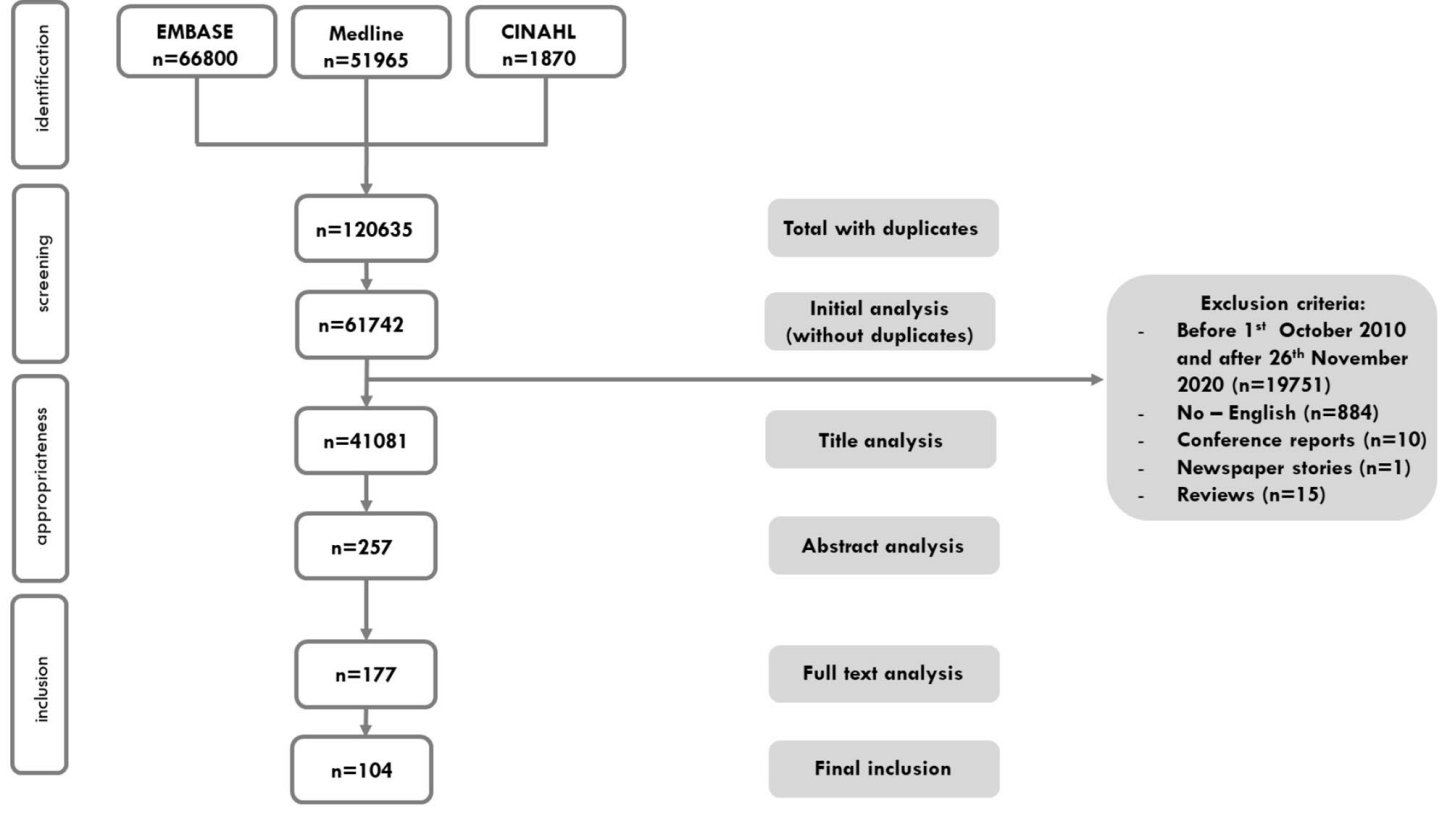
PRISMA flow diagram

Figure 1 illustrates the outcome of the search process. A total of 120,635 titles were identified after the initial search. After the removal of duplicates and title, abstract and full text analysis, 104 studies remained.

### Description of the included studies

Out of the 104 included publications, eight involved opinion papers (Hauk et al. 2018 [26], MacLeod 2014 [27], Petersen 2019 [28], Saver 2013 [29], Schrøder et al. 2019 [30], Scott et al. 2016 [15], Scott 2015 [31], Smetzer 2012 [32]), six were perspectives (Ahc 2019 [10], Tebala 2020 [33], Tamburri et al. 2017 [34], Gómez-Durán et al. 2019 [35], Shapiro et al. 2016 [36], Slykerman et al. 2019 [37]), six were editorials (Anderson et al. [38], Clarkson et al. 2019 [11], Edrees et al. 2015 [39], Everly Jr et al. 2020 [40], Thompson et al. 2015 [41], Sataloff 2020 [42]) and one was a case study (Rappaport et al. 2019 [43]). The other 83 publications were research articles (Supplementary File 1). Most studies were performed in the USA (n = 30), followed by Iran (n = 5), Canada (n = 5) and China (n = 4). More than half of the studies were observational studies and one out of four were qualitative studies. More than 9 out of 10 studies were in-hospital studies of which 25% were multi-centre studies.

### What kinds of impact of a PSI on healthcare professionals could be identified?

**Fig. 2:**
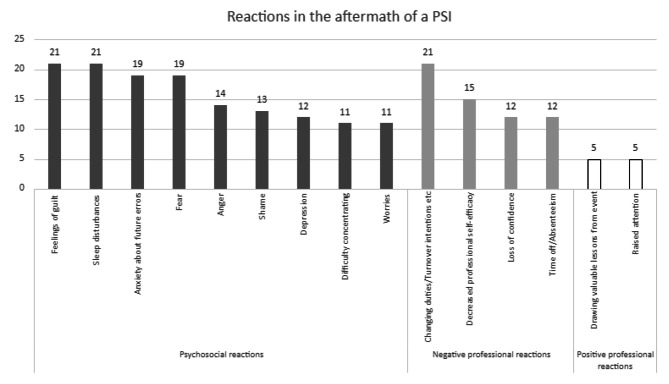
Most reported psychosocial, negative and positive professional reactions based on number of studies

Symptoms can involve psychosocial reactions, professional reactions or a physical response. The PSI could have had an impact on the mental health of the healthcare professional as a SV, leading to a wave of emotions, or a psychosocial reaction. These psychosocial reactions can for example be. feelings of guilt, sleep disturbances, anxiety or fear. Additionally, both negative and positive impacts on their way of working can emerge, Negative professional reactions can e.g. be wanting to change wards, decrease professional self-efficacy or loss of confidence. Examples of positive professional reactions include raised attention, being more careful or being more critical/self-critical. Last, physical health, mentioned by physical response have been described including e.g. eating disorder, headache or muscle tension. The details of these reactions can be found in Supplementary Files 2–5. Figure 2 provides an overview of the most commonly reported psychosocial, negative and positive reactions. Three studies mentioned that some healthcare professionals were not at all impacted by a PSI [18, 44, 45]. In contrast, other studies have posed that a PSI will always affect the involved healthcare professionals [46].

### How can healthcare professionals be supported in the aftermath of a PSI?

**Fig. 3:**
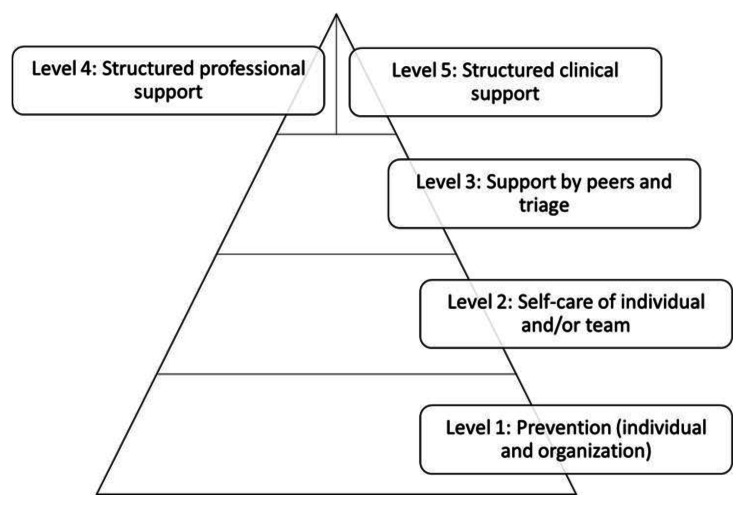
Overview of the five-level of support

For the optimal support in the aftermath of a PSI, actions should be undertaken at five -levels (Fig. 3, Supplementary File 6). The first level includes prevention on both the individual healthcare professional level and the organization level. Several actions can be taken in advance which can lead to a lower impact in the aftermath of a PSI. Examples include investments in good relationships with colleagues [47]; being a supportive and/or an active listener [26, 39, 43, 46, 48–50]; having an environment with no punitive response, where blame is avoided, an environment that is family oriented, or a just and no-macho culture [30, 39, 45, 49–58]; and being educated about the concept of the second victim and related feelings [30, 48, 50, 54, 59–61]. The second level includes self-care of the healthcare individual and team and starts when a PSI had occurred [17, 31, 36, 42, 43, 46–50, 52, 55, 57, 58, 62–81]. Some examples of support in this level are: wanting to talk in great detail about PSI/discuss the PSI [17, 46, 50, 54], trying to understand what happened and how to avoid the PSI in the future [31, 36, 43, 46, 50, 52, 73, 75], looking for support or feedback by supervisor/senior person [31, 43, 47–50, 55, 57, 63, 67–70, 82, 83]. If this level is insufficient, support by peers and triage [15, 17, 30, 31, 39, 42–50, 52–58, 60, 62–67, 69–74, 76–82, 84–92] (Level 3) can be provided, which is, if necessary, followed by structured professional support [39, 42, 43, 45–47, 49, 50, 52, 54, 56–58, 61, 64, 67, 69, 73, 76, 78–81, 87, 89, 90, 92, 93] (Level 4) and clinical support (Level 5). In level 3 (Support by peers and triage), healthcare professionals want e.g. timely support or support soon in the aftermath of a PSI [15, 31, 39, 54, 64, 87], support which is available by hotline, telephone, email, intranet [81, 85, 86] and they want to be informed about the next steps in the hospital’s process for follow-up a PSI [31, 46, 52, 60, 76, 78, 79, 90–92]. The structured professional support (Level 4), is support given by an expert in the field and can include e.g. mental health support [39, 42, 45, 52, 58, 64, 69, 73, 76, 78, 79, 93] or access to specialized support (Formal organizational support) [39, 56, 57, 92], but can also be debriefings [39, 43, 47, 50, 52, 61, 76, 78–80, 87] or mortality and morbidity meetings/clinical incident reviews [45, 50, 81, 89]. In general it treats the second victim as a person with a condition that requires e.g. short-term psychotherapy or psychosocial support, while clinical support (Level 5) treats the second victim as having a disease treatable with medication [32, 42, 44, 48, 52, 67, 73, 76, 93, 94] and long-term psychotherapy [32, 42, 44, 48, 52, 67, 73, 76, 80, 81, 93, 94].

Next to this, national and local initiatives exist that include several levels of support [37, 49, 50, 54, 56, 57, 64, 69, 73, 81, 86, 95–119] (Supplementary File 7).

### Methodological observations

Although professional and psychosocial reactions are described in many publications, only three validated questionnaires that evaluated the impact of a PSI could be identified [68, 78, 79, 91, 120–122]. First, the Second Victim Experience and Support Tool (SVEST) is increasingly being implemented as a questionnaire in second victim research [68, 69, 123]. The SVEST was originally validated in the United States and contains 29 items within seven psychosocial domains (psychological stress, physical distress, colleague support, supervisor support, institutional support, non-work-related support, professional self-efficacy) and two employment-related domains or outcomes (turnover intentions and absenteeism) [68, 121–123]. The questionnaire is available in English [69] and evaluates psychological distress, physical distress, professional self-efficacy, colleague support, supervisor support, institutional support, non-work-related support, turnover intentions, and absenteeism [68, 121–123]. Another questionnaire often applied in second victim research, is the Impact of Event Scale (IES). This evaluates the subjective distress caused by traumatic events such as PSIs [91, 120]. Last, the Second Victims im Deutschsprachigen Raum (Second Victims in German-speaking Countries) (SeViD) questionnaire was developed in Germany and Austria and covers prevalence, symptoms and support strategies of the second victim phenomenon [78, 79].

## Discussion

This is the first review focusing on preventive actions which can be taking in advance of a PSI, both by healthcare professionals and by organizations. Additionally our results showed that a difference should be made in different levels of professional support, whereby support can be either structured professionally for the SV on the short and middle term, or could be clinical, with medication or clinical-related aid on the long term. This lead to a shift of the “Scott three-tiered emotional support system towards a five-level support. These results are based on reviewing a total of 104 articles across different healthcare professionals, healthcare settings and countries. Based on the differences in published psychosocial and professional reactions, we argue that no general detailed support program is available for second victims, but that the actions should be based on the needs of the involved individual healthcare professional and should happen at different levels.

In general, the impact of a PSI on healthcare professionals is diverse, ranging from no reactions at all to psychosocial, negative and positive professional reactions, or physical reactions, which on their own could become an obstacle to patient safety. Previous studies showed that the greater the harm to the patient, the more intense the SV phenomenon is, culminating to the most severe symptoms in PSIs [22]. Healthcare organizations should also take into account that trainees and students can also become SVs in the aftermath of a PSI [62, 66, 81, 124, 125].

Only a few instruments to measure the impact of a PSI are published and the most common instrument is the Second Victim Experience and Support Tool (SVEST)., [68, 121–123] In the last two years, this instrument has been validated in several languages, e.g. Danish [126], Spanish [127], German [128]; Korean [63], Argentinian [68], Chinese [71], Italian [129], Malaysian [130] and Persian [121]. A revised version of the instrument (SVEST-R) was developed and validated in December 2020, right after the inclusion date of this review. It was first published in English [123], followed by publication of a German version [79]. The revised instrument comprises 35 items and has adapted the original domain on ‘non-work-related support’ to ‘resilience’. Additionally, seven items were added regarding desirability of second victim support options. Some of the items in the SVEST and SVEST-R questionnaire are scored inversely to reduce bias [131]. The SVEST and SVEST-R questionnaires include support and impact items by using these questionnaires in practice. This implies that the results of the questionnaire should be presented at all levels of the organization, not only on how to improve support in general to management and board, but also to supervisors and colleagues. As healthcare professionals go through different stages in their recovery [15], the SVEST or SVEST-R questionnaire should be taken at different timepoints to check the healthcare professional’s needs. This leads to the question if it is possible to translate one instrument into different languages. In other words: are these questionnaires sufficiently sensitive to capture the differences in culture, language and reactions of the healthcare professionals in the aftermath of a PSI over time? Healthcare professionals go through different stages in the aftermath of a PSI [15–17] and each stage can differ in time but can also need a different kind of support. For example, 23,6% of the involved healthcare professionals are still hypervigilant, 8,7% continue to have flashbacks, 8,2% have feelings of shame and 8,1% doubt their knowledge and skills six months after a PSI [18]. If countries, network or organizations want to set up a peer-support protocol, they should not start from scratch, but they should rely on the multilevel approach presented in this paper (see Fig. 3). However, these programs could be adapted based on their own cultural context and specific needs of the SV during the different stages of their recovery.

It is still unclear how many healthcare professionals are in need of a specific level of support according to our five-level support system (Fig. 3) in the aftermath of a PSI. Our five-level support system is an enlargement of the “Scott three-tiered emotional support system”, The “Scott three-tiered emotional support system” showed that 60% of the SVs found that the support given by colleagues and peers is very helpful and that there was no need for specially trained peer-support [20]. This review mentioned different prevention strategies which can be used by the individual healthcare professional or by the organization. A safety culture promoting support for emotional needs and increasing awareness of the SV phenomenon are seen as important preventive actions [132]. An online program such as the ‘Mitigating Impact in Second Victims (MISE) online program’ can help to improve the knowledge about PSIs, possible support models and actions in the aftermath of a PSI [133]. Professional treatment for SVs doesn’t immediately include medication or clinically-related treatment, it can also include structured professional support. However, if structured professional support is not sufficient, the next step could be treating the involved healthcare professional clinically or with medication. Interventions including visuospatial cognitive ‘Tetris’-like tasks could be implemented to reduce intrusive memories, which are common after PSIs [134]. Other methods such as cognitive processing therapy (CPT), prolonged exposure therapy (PE) and eye movement, desensitization, and restructuring (EMDR) are the current golden standards for the treatment of trauma-associated symptoms of post-traumatic stress disorder [135].

Next to learning from other SV support literature, the post-COVID era provides opportunities to enhance the SV evidence-base. A study comparing the reaction in the aftermath of a PSI by doctors and nurses with the reactions during COVID-19 showed that the impact on doctors and nurses is larger during COVID-19 than in the aftermath of a PSI [136]. Most organizations have set up a support program for their healthcare professionals during the COVID-19 pandemic, which implies that these program can be extended to SVs. Not only the level of PSIs can have an impact on the reactions, being involved in formal complaints or lawsuits can put an additional layer on the personal and professional reactions of the involved healthcare professionals [137, 138].

Some studies have shown positive reactions in the aftermath of a PSI, such as raised attention or becoming more critical. During the COVID-19 pandemic, it was shown that the pandemic can be a trigger for developing posttraumatic growth, as it is disruptive enough to affect the individual’s values and perspectives [139]. Being involved in a PSI can similarly be disruptive enough, meaning there is potential for posttraumatic growth for SVs, while for others the disruption can be a possible reason why they want to leave profession.

### Strengths and limitations

The strength of this review is that it draws together and reports all the possible reactions and support in the aftermath of a PSI. We chose to extract the reactions ad verbatim from the primary articles as to not lose subtle nuances that might depend on the language or culture of the respondents. Due to this type of research, recall bias in reactions is possible due to the fact that the symptoms were collected after the PSI had happened. These reactions were collected by (validated) questionnaires or based on qualitative research. For this reason the results of the symptoms should be interpreted by general trends and with caution. Other limitations of this review are caused by the heterogeneity of the included studies, in terms of type and severity of adverse events studied, involved healthcare professionals and types of study design. A critical appraisal of the individual studies was not performed as the aim of our study was to have a broad overview of both the reactions and support in the aftermath of a PSI.Next to this, the studies have been carried out in countries with different legal frameworks, which may affect the scope and type of intervention. Besides this, some of the studies have a small sample size or were performed at one organization which limits the generalizability of the results. Aspects of the safety culture of the individual organization should be considered when interpreting and implementing these results as differences between countries may also play a role.

### Recommendations

Further research is needed to optimize the validated instruments which measure the impact and support mechanisms for healthcare professionals in the aftermath of a PSI. This five-level support system can be used for longitudinal research but can also evaluate the impact and support needed in the aftermath of different types of PSI and different levels of support, ranging from prevention to professional and cultural support. Age, gender, professional group, working experience and personality dimensions should also be included to understand the SV phenomenon better and give the most optimal support to them. Therefore, knowledge sharing networks within and across countries should be organized. Besides measuring cultural differences, possible reactions and their duration, interventional research is needed, including randomized controlled trials to solidify different interventions at different stages of recovery and evaluate the effectiveness of the interventions.

## Conclusions

The impact of a PSI on healthcare professionals is broad, ranging from psychosocial and physical reactions to negative and positive professional reactions. This review shows that support programs should be organized at five levels. It should start with preventive actions followed by self-care of the healthcare professionals, support by peers, structured professional support and clinical support. Based on this, the involved healthcare professionals can be supported in the most optimal way in the aftermath of a PSI. Finally, organizations and healthcare professionals should take preventive actions to reduce the impact of a PSI and should provide support based on the individual needs of each healthcare professional in the aftermath of a PSI and should evolve in time based on the different stages of recovery. As each SV is a unique individual, we should be careful in implementing standard protocols or generalized approaches and provide support fitting for the SV’s personal reaction and recovering stage.

## Electronic supplementary material

Below is the link to the electronic supplementary material.

Supplementary Material 1: Additional information

## Data Availability

All data presented in this study are available in results section. Deborah Seys can be contacted if there are questions about it.
