# Supplementary material for: In search of an international multidimensional action plan for second victim support: a narrative review
Source: BMC Health Serv Res. 2023 Jul 31;23:816. doi: 10.1186/s12913-023-09637-8 (PMC10391912; doi:10.1186/s12913-023-09637-8)
Supplement: Supplementary file 1 — Supplementary Material 1: Additional information [file 12913_2023_9637_MOESM1_ESM.docx]

**Supplementary file 1** Overview of included studies

| **Study** | **Country** | **Study design** | **Respondents** | **Sample size** | **Setting** | **Timing of study** |
| --- | --- | --- | --- | --- | --- | --- |
| Abusalem et al. 2013 [1] | USA | Observational study | Home nurses | 192 | Outside hospital |  |
| Ajoudani et al. 2020 [2] | Iran | Observational study | Nurses | 298 | In-hospital (multi-centre) |  |
| Ajri-Khameslou et al. 2017 [3] | Iran | Qualitative study | Emergency nurses | 18 | In-hospital | 2014-2015 |
| Bari et al 2016 [4] | India | Observational study | Pediatric medicine residents | 130 | In-hospital |  |
| Biggs et al. 2020 [5] | Great Britain and Ireland | Observational study | Surgeons | 82 | In-hospital (multi-centre) |  |
| Brunelli et al 2018 [6] | South America | Validation study | Nurses | 169 | In-hospital |  |
| Burlison et al. 2016 [7] | USA | Observational study | Nurses | 155 | In-hospital |  |
| Burlison et al. 2017 [8] | USA | Validation study | Staff members | 305 | In-hospital |  |
| Cebeci et al. 2015 [9] | Turkey | Observational study | Nursing students | 324 | School of nursing | 2009 |
| Chan et al. 2018 [10]c | Singapore | Qualitative study | Nurses | 8 |  |  |
| Chen et al. 2019 [11] | China | Validation study | Nurses | 1442 |  |  |
| Choi et al. 2020 [12] | Korea | Observational study | Nurses | 492 | In-hospital | 2019 |
| Christoffersen et al. 2020 [13] | Norway | Qualitative study | Midwives | 33 | In-hospital (multi-centre) | 2014 |
| Chung et al. 2018 [14] | Worldwide | Qualitative study | Residents |  |  | 2016-2017 |
| Deringer et al. 2014 [15] | USA | Qualitative study | Psychiatric residents | 64 | In-hospital | 2012-2013 |
| Dukhanin et al. 2018 [16] | USA | Experimental study | Nurses, physicians, managers and others | +/-200 | In-hospital | 2011-2014 |
| Edrees et al. 2017 [17] | USA | Qualitative study | Patient safety representatives | 43 | In-hospital (multi-centre) | 2013-2014 |
| Edrees et al. 2017 [18] | USA | Qualitative study | Patient safety representatives | 43 | In-hospital (multi-centre) | 2013-2014 |
| Edrees et al. 2011 [19] | USA | Observational study | Healthcare workers | 350 | In-hospital |  |
| Edrees et al. 2016 [20] | USA | Observational study | Risk managers | 641 | In-hospital (multi-centre) | 2013 |
| Edrees et al. 2016 [21] | USA | Experimental study | Staff members | +/-500 | In-hospital | 2010-2012 |
| El Hechi et al. 2020 [22] | Canada | Experimental study | Surgeons and surgical trainees | 47 interventions | In-hospital |  |
| Finney et al. 2020 [23] | USA | Observational study | Nurses | 115 | In-hospital | 2019 |
| Graham et al. 2019 [24] | USA | Experimental study | Physicians and staff | 44 | In-hospital |  |
| Gupta et al. 2019 [25] | USA | Observational study | Physician mothers | 5782 | in-hospital | 2016 |
| Habibzadeh et al. 2020 [26] | Iran | Observational study | Nurses | 298 | In-hospital (multi-centre) | |
| Han et al. 2017 [27] | USA | Observational study | Surgeons | 126 | In-hospital (multi-centre) |  |
| Harrison et al. 2014 [28] | UK | Observational study | Physicians | 1755 |  | 2013 |
| Harrison et al. 2015 [29] | UK and USA | Observational study | Physicians and nurses | 265 | In-hospital (multi-centre) |  |
| Harrison et al. 2018 [30] | Australia and New Zealand | Observational study | Anaesthetists | 247 | In-hospital | 2016-2017 |
| Harrison et al. 2019 [31] | Vietnam | Observational study | Doctors and nurses | 497 | In-hospital |  |
| Huang et al. 2020 [32] | China | Observational and qualitative study | Nursing students | 1417 |  | 2019-2020 |
| Joesten et al. 2015 [33] | USA | Observational study | Healthcare professionals | 120 | In-hospital | 2011 |
| Kable et al. 2018 [34] | Australia | Qualitative study | Nurses | 10 | In-hospital |  |
| Kim et al. 2020 [35] | Korea | Validation study | Nurses | 305 | In-hospital | 2016 |
| Kobe et al. 2019 [36] | Canada | Observational and qualitative study | Radiation therapists | 178 |  |  |
| Koehn et al. 2016 [37] | USA | Qualitative study | Nurses | 13 | In-hospital |  |
| Krzan et al. 2015 [38] | USA | Experimental study | Pharmacy staff | 121 | In-hospital | 2013 |
| Lane et al. 2018 [39] | USA | Experimental study | Residents, fellows, faculty members, NPs/Pas and CRNAs | 165 | In-hospital | 2014-2017 |
| Luu et al. 2012 [40] | Canada | Qualitative study | Surgeons | 20 |  |  |
| McCarthy et al. 2016 [41] | Ireland | Experimental study | Junior doctors | 208 |  | 2011-2013 |
| McLennan et al. 2015 [42] | Switzerland | Observational study | Anaesthesiologist | 281 | In-hospital (multi-centre) | 2012-2013 |
| Merandi et al. 2017 [43] | USA | Qualitative study | Healthcare workers | 232 | In-hospital | 2012-2016 |
| Merandi et al. 2018 [44] | USA | Experimental study | Nurses, APN, therapist, unit clerk, physician, manager, patient care assistant, unit technician, dietician, pharmacist, service coordinator, other | 250 | In-hospital | 2016 |
| Mira et al. 2015 [45] | Spain | Observational study | Doctors, nurses other | 1087 | In -and out-hospital |  |
| Mira et al. 2015 [46] | Spain | Observational study | Managers and patient safety coordinators | 406 | In -and out-hospital | 2014 |
| Mohamadi-Bolbanabad et al. 2019 [47] | Iran | Observational study | Physicians, nurses and midwives | 338 | In-hospital | 2017 |
| Mohsenpour et al. 2018 [48] | Iran | Qualitative study | Nurses | 8 | In-hospital (multi-centre) |  |
| Mok et al. 2020 [49] | Singapore | Observational study | Nurses | 1163 | In-hospital | 2018 |
| Panella et al. 2016 [50] | Italy | Observational study | Physicians | 1313 | In-hospital | 2014 |
| Pijl Zieber et al. 2015 [51] | Canada | Qualitative study | Nursing students | 16 |  |  |
| Pinto et al. 2014 [52] | UK | Observational study | General and vascular surgeons | 47 | In-hospital |  |
| Pratt et al. 2015 [53] | USA | Qualitative study | Panel of stakeholders | 14 |  |  |
| Pyo et al. 2020 [54] | Korea | Observational study | Physicians | 895 |  |  |
| Quillivan et al. 2016 [55] | Columbia | Observational study | Nurses | 155 | In-hospital | 2013 |
| Reiser et al. 2020 [56] | Switzerland | Observational study | Hospitals’ quality managers and chief executive officers | 116 | In-hospital | 2018 |
| Rinaldi et al. 2016 [57] | Italy | Qualitative study | Nurses, physicians and other healthcare workers | 33 | In-hospital | 2012-2013 |
| Scheepstra et al. 2020 [58] | The Netherlands | Observational study | Gynaecologists, orthopaedic surgeons, pediatricians | 1374 | In-hospital |  |
| Schrøder et al. 2019 [59]s | Denmark | Observational study | Midwives and obstetricians | 2098 |  | 2012 |
| Stangierski et al. 2012 [60] | Poland | Observational study | Doctors | 100 |  |  |
| Stone 2019 [61] | USA | Qualitative study | Nurses |  |  |  |
| Stone 2020 [62] | USA | Qualitative study | Nurses | 12 | In-hospital |  |
| Strametz et al. 2021 [63] | Germany | Validation study | Nurses, physicians, medical assistants, physician assistants, paramedics, medical therapists and remedial therapist | 306 | In- and out-hospital | 2020 |
| Strametz et al. 2021 [64] | Germany | Observational study | Physicians | 555 | In-hospital | 2019 |
| Stukalin et al. 2019 [65] | Canada | Observational study | Physicians | 51 |  |  |
| Swartwout et al. 2017 [66] | USA | Validation study | Nurses | 497 |  |  |
| Tan et al. 2020 [67] | China | Validation study | Nurses | 731 |  |  |
| Treiber et al. 2018 [68] | USA | Observational study | Nursing graduates | 168 |  |  |
| Tumelty 2018 [69] | Ireland | Qualitative study | Medical training and legal professionals | 18 |  | 2015 |
| Ullström et al. 2014 [70] | Sweden | Qualitative study | Healthcare professionals | 21 | In-hospital | 2011 |
| Van Gerven et al. 2016 [71] | Belgium | Observational study | Physicians, nurses and midwives | 913 | In-hospital |  |
| Van Gerven et al. 2016 [72] | Belgium | Qualitative study | Physicians, nurses and midwives | 31 |  | 2012-2013 |
| Van Gerven et al. 2016 [73] | Belgium | Observational study | Physicians and nurses | 5788 | In-hospital |  |
| Vanhaecht et al. 2019 [74] | The Netherlands | Observational study | Doctors and nurses | 4369 | In-hospital |  |
| Vinson et al. 2014 [75] | USA | Observational study | Anaesthesia residence |  | In-hospital |  |
| Wahlberg et al. 2020 [76] | Sweden | Qualitative study | Midwives and obstetricians | 14 | In-hospital |  |
| White et al. 2015 [77] | USA | Observational study | US members of ASHRM | 635 |  | 2013 |
| Winning et al. 2018 [78] | USA | Observational study | Healthcare providers in neonatal intensive care unit | 463 | In-hospital | 2015 |
| Winning et al. 2020 [79] | USA | Validation study | Healthcare providers in neonatal intensive care units | 514 | In-hospital |  |
| Yung et al. 2016 [80] | Taiwan | Observational study | Nurses | 306 | In-hospital |  |
| Zeeman et al. 2020 [81] | The Netherlands | Observational study | Nurses and doctors | 2635 | In-hospital (multi-centre) | 2016-2017 |
| Zhang et al. 2020 [82] | China | Validation study | Nurses | 625 | In-hospital (multi-centre) |  |
| Zhang et al. 2019 [83] | China | Observational study | Nurses | 267 | In-hospital (multi-centre) | 2017 |

**Supplementary file 2 :** Psychosocial reactions

| **Psychosocial reactions** | **Number of times reaction used in literature** | **References** |
| --- | --- | --- |
| Feelings of guilt | 21 | [1, 4, 5, 9, 25, 27, 29, 36, 45, 48, 63, 64, 68, 70, 72, 76, 80, 84-87] |
| Sleep disturbances | 21 | [11, 12, 25, 28, 30, 34, 36, 42, 45, 47, 49, 54, 57, 63, 64, 70, 72, 74, 81, 84, 86] |
| Anxiety about future errors | 19 | [9, 17, 19, 27, 28, 30, 34, 40, 42, 45, 48, 52, 58, 68, 70, 72, 79, 84, 87] |
| Fear | 19 | [9, 11, 32, 34, 36, 48, 49, 51, 52, 57, 61, 68, 72, 74, 80, 81, 84, 86] |
| Anger | 14 | [1, 25, 27, 30, 34, 36, 57, 63, 64, 68, 72, 84, 87, 88] |
| Shame | 13 | [11, 25, 27, 32, 34, 66, 68, 70, 72, 74, 84, 87] |
| Depression | 12 | [19, 34, 36, 47, 57, 58, 63, 64, 70, 72, 84] |
| Difficulty concentrating/Unable to concentrate | 11 | [36, 45, 47, 57, 62-64, 66, 72, 84, 86] |
| Worries | 11 | [5, 9, 11, 28, 29, 32, 33, 36, 48, 66, 70] |
| Psychological distress | 9 | [8, 23, 32, 35, 49, 58, 74, 82, 83] |
| Sadness | 9 | [6, 9, 25, 27, 36, 57, 70, 72, 87] |
| Remorse | 8 | [1, 3, 32, 36, 48, 49, 57, 68] |
| Looking for personal reassurance | 8 | [6, 24, 34, 36, 68, 70, 84, 89] |
| Doubts | 8 | [29, 34, 36, 57, 61, 74, 81] |
| Repetitive memories | 7 | [45, 47, 61, 62, 70, 89, 90] |
| Frustration | 7 | [36, 47, 57, 68, 70, 72, 84] |
| Flashback | 7 | [34, 57, 63, 64, 72, 74, 81] |
| Isolate by silence | 6 | [20, 34, 48, 62, 72, 84] |
| Substance abuse | 6 | [34, 52, 63-65, 73] |
| Stress | 5 | [34, 40, 72, 81, 84] |
| Experiencing burnout | 5 | [17, 25, 72, 73, 79] |
| Traumatic stress | 5 | [34, 52, 54, 58, 90] |
| Scared | 5 | [28, 29, 62, 68, 72] |
| Self-blame | 5 | [34, 52, 65, 72, 88] |
| Generally distressed | 4 | [28-30, 48] |
| Shock | 4 | [34, 52, 70, 72] |
| Tension in relationship at home (with children or partner) | 4 | [5, 51, 72, 86] |
| Sense of failure | 3 | [34, 40, 84] |
| Hypervigilance | 3 | [12, 74, 81] |
| Regret | 3 | [29, 36, 80] |
| Shame | 3 | [36, 63, 64] |
| Generally anxious | 3 | [28-30] |
| Being fearful about being blamed, losing their registration, being punished, being investigated, losing their job | 3 | [34, 63, 64] |
| Resilience | 3 | [51, 72, 79] |
| Panic | 3 | [34, 72, 76] |
| Disappointment | 3 | [5, 68, 72] |
| Disbelief | 3 | [34, 70, 72] |
| Helplessness | 3 | [34, 36, 72] |
| Extreme/excessive fatigue | 3 | [36, 57, 84] |
| Exhaustion | 3 | [34, 48, 72] |
| Dejected | 3 | [72, 74, 81] |
| Emotional distress | 2 | [4, 6] |
| Became nervous | 2 | [66, 68] |
| More irritable | 2 | [72, 84] |
| Restlessness | 2 | [3, 80] |
| Crying | 2 | [68, 72] |
| Grief | 2 | [34, 36] |
| Desperation | 2 | [72, 86] |
| Fear of reputation damage | 2 | [36, 57] |
| Fear of blame | 2 | [34, 86] |
| Being horrified | 2 | [52, 68] |
| Relief | 2 | [68, 80] |
| Having negative feelings | 2 | [34, 72] |
| Finding self-identity | 2 | [10, 34] |
| Having intrusive thoughts (feeling disheartened, having fear of making the same mistake, wondering how they could make such a mistake) | 2 | [10, 34] |
| Punitive stance | 2 | [52, 65] |
| Abandon patient and simply run away = “get-away” reaction | 2 | [76, 86] |
| Behavioral disengagement | 2 | [52, 65] |
| Venting | 2 | [52, 65] |
| Social isolation | 2 | [63, 64] |
| Angry | 1 | [1] |
| ‘I hate that I made a medication error’ | 1 | [68] |
| Humiliation | 1 | [84] |
| Disheartened | 1 | [72] |
| Embarrassment about needing psychological support | 1 | [33] |
| Ashamed by the error | 1 | [66] |
| Negative towards yourself | 1 | [30] |
| Responding psychically after the event | 1 | [10] |
| Fearful of future occurrences | 1 | [49] |
| Afraid another mistake was going to occur again | 1 | [61] |
| Afraid of reporting error | 1 | [68] |
| Excessive excitability | 1 | [57] |
| Upset with myself | 1 | [66] |
| Dwelling in their memories of it | 1 | [48] |
| Trouble memories | 1 | [33] |
| Reflection on the details of the case and alternative pathways of rumination on ‘what might have been’ | 1 | [40] |
| Recurring thoughts about error | 1 | [66] |
| Anxiety about being able to continue practice such as a commitment to providing support to families affected by adverse event | 1 | [34] |
| Anxious to even type out what happened | 1 | [68] |
| Extreme anxiety about disclosing to the patient and/or family | 1 | [33] |
| Anxiety for patient’s future | 1 | [48] |
| Uneasiness | 1 | [34] |
| Sorrow | 1 | [4] |
| External blame undermines self-confidence | 1 | [86] |
| Fear of the unknown | 1 | [84] |
| Fear for future (litigation, reoccurrence of the same incident) | 1 | [72] |
| Often preferred to be punished as they could relax and restore their lives afterward | 1 | [48] |
| Fear to perform high risk procedures | 1 | [11] |
| Fear for loss of reputation | 1 | [72] |
| Fear about their clinical performance | 1 | [52] |
| Despite the caution and fear from her previous mistake experience, she pushed herself to confront her fears and made herself go back into a similar acute-care rotation | 1 | [51] |
| Worries about disciplinary action | 1 | [70] |
| Worries about job loss | 1 | [70] |
| Worry about lawsuit | 1 | [33] |
| Worried about what people would think of them | 1 | [33] |
| Concerned about what people would think of me | 1 | [66] |
| Concern about error over time | 1 | [66] |
| Self-consciousness | 1 | [3] |
| loss of consciousness | 1 | [48] |
| Constantly holding yourself accountable | 1 | [61] |
| Feeling relieved that the harm impact was minimal | 1 | [34] |
| Happy patient was not harmed | 1 | [68] |
| Was grateful that the patient was okay | 1 | [68] |
| Being stunned | 1 | [52] |
| Felt awful | 1 | [68] |
| Taking on the blame | 1 | [76] |
| Self-recrimination | 1 | [80] |
| ‘I was mad and disappointed at myself’ | 1 | [68] |
| Feeling a reluctance to admit the severity of the adverse event | 1 | [40] |
| Heavy mood | 1 | [80] |
| Felt like terrible nurse after making error | 1 | [66] |
| Miserable | 1 | [11] |
| Unhappy | 1 | [29] |
| Deeply accusated | 1 | [11] |
| Being accused: blame and compassion offered by colleagues, patients, judges and others | 1 | [48] |
| Found themselves in court of conscience (accused, judge and victim) | 1 | [48] |
| Preoccupied with judging their own culpability | 1 | [48] |
| Affliction/displeasure | 1 | [57] |
| Tensed | 1 | [72] |
| Change in personality | 1 | [72] |
| ‘Terrified I may have harmed a patient’ | 1 | [66] |
| ‘Mortified I made an error’ | 1 | [66] |
| ‘Felt I let my patient down’ | 1 | [66] |
| Troubling thoughts about nursing error | 1 | [66] |
| Mind is always busy with negative thoughts and feelings about life of patient | 1 | [86] |
| Did not trust themselves | 1 | [34] |
| Feeling used | 1 | [62] |
| Over-reaction | 1 | [34] |
| Need to exaggerate these negative emotions during this time as they believed they should be punished for the event | 1 | [40] |
| Beat themselves up | 1 | [34] |
| Vulnerability | 1 | [34] |
| Long-term effects of making an error lingered | 1 | [68] |
| Positioning reframing | 1 | [52] |
| Mistakes vicariously | 1 | [52] |
| Restore personal integrity and endure the inquisition | 1 | [40] |
| Moral and ethical reasoning and confusion that they experienced | 1 | [52] |
| Spiritual crisis | 1 | [51] |
| Tendency to imbue themselves with an overwhelming sense of being wrong | 1 | [51] |
| Disintegrating sense of self | 1 | [51] |
| Once their sense of self started to disintegrate it became very difficult to regain their confidence | 1 | [51] |
| Highly negative self-image | 1 | [89] |
| Suffering a mental block | 1 | [76] |
| Self-forgiveness | 1 | [91] |
| Tiredness | 1 | [45] |
| Facing the horror of losing control | 1 | [76] |
| Error was important to them and that it had created an obsession in them | 1 | [48] |
| Personal reaffirmation | 1 | [89] |
| Realization of imperfection | 1 | [72] |
| Powerlessness | 1 | [72] |

**Supplementary file 3:** Positive professional reactions

| **Positive professional reactions** | **Number of times reaction used in literature** | **References** |
| --- | --- | --- |
| Drawing valuable lessons from event (learning from the mistake, sharing personal experiences, taking extra precautions) | 5 | [10, 34, 51, 68, 83] |
| Raised attention | 5 | [1, 3, 29, 37, 40] |
| Become more careful / Attention for prevention measures | 3 | [40, 70, 72] |
| More critical /self-critical | 2 | [70, 72] |
| Planning | 2 | [52, 65] |
| More valued relationship with colleagues | 2 | [29, 51] |
| Taking responsibility for the mistake made (accepting the consequences, acknowledging mistakes made by others/self, making amends) | 2 | [10, 34] |
| More confident in abilities | 2 | [30, 78] |
| Necessary part of learning about how to be a nurse | 1 | [37] |
| Continue to remember and be impacted by the event in the larger context of making something positive come from the error experience | 1 | [91] |
| Higher awareness of responsibility | 1 | [72] |
| Positive reframing | 1 | [65] |
| Controllability of the causes that led to the incident | 1 | [52] |
| Take responsibility and ownership | 1 | [86] |
| Act professionally | 1 | [86] |
| ‘Learn something’ that would change future practice and benefit future patients | 1 | [40] |
| ‘Good’ needed to come from the event and thus learning for the next was an essential step in ‘moving on’ | 1 | [40] |
| Being powerless but responsible | 1 | [76] |
| Feeling determined | 1 | [29] |
| Alert | 1 | [29] |
| Increased attention to safety issues | 1 | [29] |
| Use of evidence based medicine | 1 | [4] |
| More training than ever before | 1 | [3] |
| Enhancing skill | 1 | [3] |
| Searching information and seeking consultation | 1 | [3] |
| Stating the error and sharing one’s experiences | 1 | [3] |
| Opportunity to learn | 1 | [48] |
| Improved their accountability | 1 | [48] |
| More sensitive about their profession after making an error and more concerned ever about their responsibility | 1 | [48] |
| Check everything | 1 | [34] |
| On term effects in clinical practice including attending training to improve clinical skills | 1 | [34] |
| Determined to improve | 1 | [30] |
| Personally, confirming data | 1 | [1] |
| Changing organization of data | 1 | [1] |
| Opportunity to take time out from clinical duties and access counseling | 1 | [33] |
| Taking leave from work | 1 | [29] |

**Supplementary file 4 :** Negative professional reactions

| **Negative professional reactions** | **Number of times reaction used in literature** | **References** |
| --- | --- | --- |
| Changing duties/wards/units or job/turnover intentions /career choice /second guessing their career | 21 | [6, 8, 11, 12, 23, 25, 34, 35, 38, 49, 57, 58, 61, 73, 78, 82-84, 86] [54, 72] |
| Decreased professional self-efficacy | 15 | [2, 6, 8, 19, 23, 32, 35, 49, 55, 57, 67, 78, 82, 83, 92] |
| Loss of confidence | 12 | [9, 25, 28, 30, 34, 45, 47, 57, 63, 64, 66, 84] |
| Time off/Absenteeism | 12 | [6, 8, 25, 33, 35, 45, 49, 63, 64, 68, 82, 83] |
| Decreased job satisfaction | 7 | [28, 30, 31, 36, 42, 47, 57] |
| Defensive medicine | 7 | [4, 50, 58, 63, 64, 72, 88] |
| Fear of reputation damage | 4 | [28, 30, 42, 47] |
| Anxiety/doubting to go back to work | 4 | [34, 36, 57, 72] |
| Felt incompetent in my nursing skills | 3 | [51, 66, 89] |
| Affected relationship with colleagues | 3 | [5, 28, 30] |
| Avoiding risks | 3 | [52, 74, 81] |
| Avoid caring for similar patients | 2 | [1, 57] |
| Ability to perform job | 2 | [19, 42] |
| Feeling excluded | 2 | [76, 86] |
| Feeling others’ prejudice (being labeled, enduring gossip) | 2 | [10, 34] |
| Blocked (not being able to perform profession anymore) | 2 | [17, 72] |
| Aggressive/risky behaviour | 2 | [63, 64] |
| Loss of self-confidence in nursing skills | 2 | [66, 72] |
| Feeling uncomfortable within team | 2 | [74, 81] |
| Feeling unable to provide quality care | 2 | [74, 81] |
| Keeping error for themselves | 1 | [1] |
| Felt less able to work safely and effectively | 1 | [33] |
| Concern about perceived professional incompetence | 1 | [34] |
| Treated differently following the mistake | 1 | [51] |
| Instructor always quizzing her and was always on top of her | 1 | [51] |
| Being felt left out | 1 | [62] |
| Concerns about devastating loses for families, patients in critical states and colleagues in involved events | 1 | [34] |
| Concern about how their actions might be viewed by their colleagues and managers and might criticize their actions and perceive them as incapable of doing their job adequately or appropriately | 1 | [34] |
| Worry that coworkers are discussing her or his professional performance | 1 | [84] |
| Strained colleague relationship | 1 | [29] |
| They felt critized when they make them | 1 | [68] |
| Medico-legal implications | 1 | [86] |
| Professional concerns about being investigated | 1 | [34] |
| Feelings of professional inadequacy | 1 | [36] |
| Permanent reduction clinical role | 1 | [25] |
| See fewer patients | 1 | [4] |
| Face long-term career sequelae | 1 | [84] |
| Jeopardize their careers | 1 | [84] |
| Future employment | 1 | [84] |
| Reason for earlier retirement | 1 | [40] |
| ‘Concerned I may lose my job’ | 1 | [66] |
| Recovery from the error event typically leads the clinician to do some significant soul searching about whether to drop out, survive or thrive. Dropping out includes either leaving the medical profession or making a change in locale | 1 | [91] |
| Voluntarily stepped down as unit’s charge nurse | 1 | [61] |
| Change scope or type of their practice because they could not effectively cope with their reactions to adverse events and had chosen instead a ‘safer’ alternative | 1 | [40] |
| Compassion satisfaction | 1 | [79] |
| Bad work performance | 1 | [11] |
| Decline in clinical performance | 1 | [84] |
| Negative work-related outcome | 1 | [35] |
| Doubted professional judgment and sometimes career choice | 1 | [70] |
| Loss of trust in hospital and/or medicine | 1 | [72] |
| Uncertainty | 1 | [72] |
| Questioning actions and skills | 1 | [62] |
| Feeling insecure in professional roles | 1 | [70] |
| Become increasingly cautious with future patients | 1 | [34] |
| Collapse in false confidence about their knowledge and power | 1 | [48] |
| Experiencing uncertainty in the inquiry | 1 | [34] |
| Having competing duties | 1 | [76] |
| Loss of delight of work | 1 | [72] |
| Normal process of their clinical activities suddenly “went sideways” | 1 | [52] |
| Concerned about documentation | 1 | [34] |
| Became obsesses in my nursing practice after event | 1 | [66] |
| Wanted to be accurate and correct in their learning and in their clinical performance, but they adopted the notion that they must be wrong | 1 | [51] |
| The term “lurk” reflects those participants did not often discuss the memories of the error and the feelings associated with it, but these memories and feelings remained under the surface and continued to haunt them. Because the experience of the error lurked in the minds of the participants, they maintained an enduring vigilance to ensure a similar error did not occur again. In this sense, lurking could have a positive connotation. | 1 | [37] |
| Suffering from negative reaction from their supervisors | 1 | [89] |

**Supplementary file 5 :** Physical reactions

| **Physical reactions** | **Number of times reaction used in literature** | **References** |
| --- | --- | --- |
| Physical stress/physical distress | 8 | [8, 23, 35, 49, 55, 66, 82, 83] |
| Eating disorder/Eating disturbance/Loss of appetite | 7 | [11, 12, 49, 54, 72, 84, 86] |
| Nausea (when exposed to similar situation) | 6 | [11, 12, 49, 68, 72, 84] |
| Gastralgia | 3 | [47, 57, 72] |
| Headache | 3 | [47, 57, 84] |
| Muscle tension | 3 | [36, 57, 84] |
| Tachycardia | 2 | [47, 57] |
| Cold sweat when exposed to similar situation | 2 | [12, 54] |
| Psychosomatic reactions | 2 | [63, 64] |
| Losing weight | 1 | [72] |
| Body tension when exposed to similar situation | 1 | [12] |
| Psychosomatic distress | 1 | [67] |
| Increased blood pressure | 1 | [57] |
| Increased respiratory rate | 1 | [57] |
| Shaking (across entire body and sweating hands) | 1 | [72] |
| Illness | 1 | [72] |
| Devastation | 1 | [34] |
| Experienced vertigo, dyspnea, cold sweat or muscle rigidity when exposed to similar situation | 1 | [54] |

**Supplementary file 6**: Levels of support

| **Level 1: Prevention as individual or as organization** | |
| --- | --- |
|  | - Invest in good relationships with colleagues [59] - Talking openly your feelings [93] - Feeling of being part of the workplace [59, 94] - Be supportive/active listener [16, 36, 57, 70, 89, 94, 95] - Take responsibility for PSI [72] - Supportive environment/open setting to discuss problems/PSI [21, 61, 70] - Provide good atmosphere/teamwork between colleagues [56, 59] - Provide good co-operation between colleagues [59] - Work together as a team [34, 44, 48] - Be respectful [94] - Combination of support and atmosphere [57] - Willing to listen to work related problems/belief them [59, 61, 95] - Not negatively judging [16, 42, 59, 70, 77, 94] - Give sympathy and attention [48] - Support by unit/own department [16, 44, 45, 75] [63, 64] - Outreach designed with “opt-out” instead of “opt-in” strategy [22] - Education of peers should include role playing with critiques by observer experts[17, 22, 44] - Share best practices (psychological support useful) [21, 42, 77] - Provide to take time out from clinical duties in the aftermath of a PSI [8, 18, 21, 42, 44, 63, 64, 77] - Support staff to feel competent to support SV in aftermath of PSI [20] - Provide additional guidance at workplace [71] - Learning opportunities/locus of causes that led to PSI/supportive learning environment [13, 33, 51-53, 62, 68, 95, 96] - Opportunity for SV to “stop and reground instead of being “robot mode” [68] - No punitive response/environment /avoid blame/family oriented environment/just culture/No macho culture [5, 16, 21, 27, 31, 33, 36, 44, 49, 52, 68, 85, 95] - Take appropriate steps to prevent PSI [18, 21, 33, 59, 63-65, 95] - Reduce Stigma[21] - No additional cost for SV [62] /look for sustainable funding[16, 21, 77, 97] - Provide one-to-one support format [13, 16, 17, 94] - Provide group format [14, 16-18] - Provide structure and routine for handling PSI and support staff [21, 70] - Educate about SV/feelings /training needs[14, 20, 21, 36, 53, 57, 85] - Educate peers [14, 22] - Understand that system errors are results of system and not result of individuals fault [21, 76] - Managers: support/empathy [5, 8, 11, 17, 20, 21, 24, 31-33, 36, 49, 59, 70, 77] - Support by organization [2, 5, 8, 16, 23, 35, 65, 67, 70, 78, 83] - Provide peaceful location [8, 49, 78] - Follow-up PSI [16, 22, 62, 70] - Possibility to report any concerns about patient safety without fear of retribution or punitive action [31, 33] - Information how to access confidential emotional support within institution if needed [17, 33, 59] - Information about contact person [46] - Visibility of professional help/support service [62, 70] - Encourage open dialogue about SV [91] - Include enough peer supporters [44] - Attention for well-being of healthcare professionals [49] - Open communication to help to build trust in organization and its staff [46] |
| **Level 2: Self-care of individual and team** | |
|  | - Non-work related support (partner/friends/family/GP) [6, 8, 11, 16, 19, 27, 31-36, 40, 49, 51, 57, 59, 62, 67, 70, 78, 82, 83, 87] - Try to understand what happened and how to avoid it [33, 34, 36, 37, 70, 84, 89, 93] - Support by colleague involved in (previous) PSI [5, 98] - Support/feedback by supervisor/senior person [1, 6, 8, 15, 16, 19, 31, 35, 36, 49, 57, 59, 83, 84, 89] - Talk to patient and/or family [1, 5, 16, 28, 33, 40] - Formal support [18, 28, 36, 63, 64, 89] - Informal support [18, 28, 34, 36, 62-64, 89] - Feeling of not being alone [24] - Needing to talk in great detail about PSI/discuss the PSI [21, 36, 40, 70] - Looking for confirmation of actions and decisions [70] - Looking for info that would alleviate their feelings of failure [40] - Mentoring/supportive guidance [5, 18, 31] - Provide immediately support [59] - Be empathic [36, 70] - Collegial trust [34] - Analyze what has happened and alleviate the pressure SV feel [46, 71] - Offer empathy and share personal experiences [22] - Review common symptoms and feelings clinicians experience [22] - Reframe event [93] - Confirm how the SV can carry out their work [59] - Support actively participate to work through PSI [63, 64] |
| **Level 3: Peer support and triage** | |
|  | - Support by colleagues/peers (discussion/interaction/empathy) [1, 5, 8, 11, 16, 19, 21, 23, 27, 28, 31, 32, 34-36, 40, 43, 44, 49, 51, 57, 59, 62, 67, 68, 70, 78, 82-84, 87, 89, 98] - Confirm that the SV did the right thing [24] - Give SV space to vent [24] - Give more supervision [5] - Supervisor: activate peer support program [44] - Seniors: give advice to SV [5] - Available by hotline, telephone, email, intranet [22, 43, 77] - Timely support/support soon in aftermath of PSI [17, 21, 62, 84, 95, 98] - Confidential way to get in touch with someone 24hr a day [8, 16, 21, 42, 49, 77] - Anonymity [16] - Discuss PSI with colleagues to prevent them from repeating error [48, 95] - Arrange peer support program [85] - Department based support program [75] - Residency/Resident based support program [75] - Peer support [70] - Peer supporter should adopt well established strategy when engaging affected peer [22, 84] - Supported in how to disclose PSI to patient and/or family [33] - Personal legal advice and support [18, 33, 63, 64] - Inform about the next steps in the hospital’s process for follow-up a PSI [18, 33, 46, 53, 63, 64, 70-72, 84] - Opportunity to discuss emotional and ethical issues [63, 64] - Communication/Needs of patient and/or family after PSI were appropriately met [18, 33, 59, 63, 64] |
| **Level 4: Structured professional support (treated as condition)** | |
|  | - Support by pastoral care services/chaplain [17, 19, 44, 62] - Support by neutral person [16] - Mental health support/(professional)counselling/coaching [5, 8, 18, 25, 27, 33, 34, 62-64, 87, 95] - Debriefings [14, 17, 18, 24, 33, 36, 59, 63, 64, 89, 95] - Crisis intervention [33, 36] - Mortality and morbidity meetings/clinical incident reviews [5, 22, 36, 75] - Provide access to specialized support (formal organizational support) [44, 46, 49, 95] - Root cause analysis [21, 24, 33, 36, 62, 70, 72] - Give time to schedule time with counsellor to discuss event[49] |
| **Level 5: Clinical support (medication related/more clinical support)** | |
|  | - Support by psychologist/psychiatrist [18, 19, 25, 33, 34, 42, 45, 57, 87, 96] - HEAR consult team [24] - Possible need for continued support [22] - Should also include outside service [44] |

**Supplementary file 7** Details of different second victim programs

| Name of programme | Institution | Country | Core elements |
| --- | --- | --- | --- |
| Resilience in Stressful Events (RISE) [16, 99] | Johns Hopkins | USA | - Increase awareness of second victim phenomenon - Provide multi-disciplinary, one-on-one or group, peer support in a non-judgemental environment - Equip managers and employees with healthy coping strategies to promote well-being - Reassure and guide employees to continue thriving their roles |
| Center for Professionalism and peer support (CPPS) [100, 101] | Royal Brisbane and Women’s Hospital | USA | - Briefing conversation before meeting with patient and family - Helpful resources: risk management, trained clinician disclosure coaches, division chief/chair, other healthcare team members and patient/family relations |
| the Adverse Event Response Team [AVERT] Program[102, 103] | Physicians Insurance | USA | N/A |
| Institute for Healthcare Improvement[104] | Institute for Healthcare Improvement | USA | N/A |
| Care of the Caregiver, an endorsed safe practice[105] | National Quality Forum | USA | The involved caregivers should receive timely and systematic care to include: treatment that is just, respect, compassion, supportive medical care, and the opportunity to fully participate in event investigation and risk identification and mitigation activities that will prevent future events |
| Schwartz center[106] | Schwartz center | USA | Research-based strategies, tools and support that caregivers need to create and sustain cultures of compassion |
| Medically Induced Trauma Support Services (MITSS) [107] |  | USA | - assessment of internal culture of safety - organizational awareness of adverse events and response of clinicians and staff - formation of multi-disciplinary advisory group - leadership buy-in from senior administrative team - risk management considerations regarding rapid disclosure and support - policies, procedures and practices regarding handling of adverse events and crisis management - operational core element is defined by determining who/what/when/how to activate support mechanism - dissemination/communication plan to increase awareness and educate employees on all levels - learning and improvement opportunities for development of strategies to continually evaluate and improve support program |
| The second victim[108] | Institute for Safe Medication Practices (ISMP) | Canada | Healthcare practitioners:   - whenever possible, participate in disclosure - be a part of the solution - seek help from your organization or from peers - share your story   Healthcare organizations:   - ensure staff are aware of and can access organizational support programs - strong peer support is emerging as being especially helpful - involve the healthcare practitioner in the disclosure process and in the development of system solutions |
| Novel surgery-specific Second Victim peer support program[22] | Massachusetts General Hospital | USA | - create a conceptual framework - choice of peer supporters - training of peer supporters - multi-faceted identification of major adverse events - design of a systematic intervention plan |
| forYOUteam [109] | Missouri Health Care | USA | - provide “emotional first aid” to our staff who have been involved in unanticipated or stressful events - provide one-on-one peer support and explore the staff member’s normal reactions and feelings that often occur after a stressful or traumatic event - provide the second victim with a “safe zone” to express thoughts and reactions to enhance coping - provide the employees assurance that he or she is experiencing a normal reaction - ensure that the sharing of information is strictly confidential |
| Second victim program[110] | Nationwide Children’s Hospital | USA | Based on Scott Three-Tiered interventional Model of Second victim support:   - Local (unit/department) support - Trained peer support - Expedited referral network |
| KP Care for Caregivers Toolkit ^[111]^ | Kaiser Permanente | USA | - Awareness-Raising Resources - Tools for leaders supporting healthcare providers (leadership guidelines/tips for leadership) - Information for healthcare providers (coping with traumatic events/six stages of recovery) |
| Trust Team [112] |  |  | - Treatment that is fair and just - Respect - Understanding and compassion - Supportive care - Transparency and opportunity to contribute |
| Clinical Peer Support program (PSP) [39] | Barnes-Jewish Hospital (JWH) and its affiliated hospital St. Louis Children’s Hospital (SLCH) | USA | - Training (Evidence-based information on emotional and functional impact of adverse events/medical errors for involved clinicians) - Peer support clinicians - Set up program structure |
| Second victim program[113] | KU Leuven | Belgium | - Practical information - Education of healthcare professionals - Emotional support (immediately/support team/psychotherapy)(based on Scott Three-Tiered Interventional Model of second victim) |
| Institute for Patient Safety Excellence [114] | University of Illinois (Chicago) | USA | N/A |
| EAP (Employee assistance program) [8, 21, 34, 36, 44, 49, 62, 77] | Not disclosed | USA | N/A |
| Patient safety online programme for doctors [41] | Royal College of Physicians of Ireland | Ireland | - Introduction to patient safety - Understanding adverse events and near misses - Open communication - Caring for the second victim - Learning from adverse events and near misses |
| Curriculum for Certified Registered Nurse Anaesthetists (CRNAs)[115] | Not disclosed | USA | - Define and describe second victim - Second victim risks for nurse anaesthetists - Barriers for second victims - Consequences for the second victim - Evidence-based understanding and intervention frameworks - Support systems |
| Patientensicherheit Schweiz[116] | Swiss Patient Safety Foundation | Switzerland | N/A |
| Helping Others Process the Event (HOPE) [117] | Not disclosed | North Carolina | N/A |
| ANZCA Doctors’ Support Program[118] | Australian and New Zealand College of Anaesthetists | Australia and New Zealand | - 24/7 support - Critical Incident Debriefing Toolkit - Doctors’ health advisory services |
| Catastrophes in Anaesthetic Practice-dealing with the aftermath [119] | Association of Anaesthetists of Great Britain and Ireland (AAGBI) | UK and Ireland | - Actions to take after event - Role of anaesthetic department - Trust response - How a catastrophe may affect you personally - Critical incident stress debriefing (CISD) - Medico-legal issues |
| Critical Incident Reporting System (CIRS) [120] | Not disclosed | Not disclosed | N/A |
| MISE (mitigating impact in second victims)[121] | Not disclosed | Spain | - awareness-raising on the impact of the second victim phenomenon - preventive approach to reduce the impact of highly stressful situations such as errors with serious consequences |
| International Critical Incident Stress Foundation[122] | Not disclosed | USA | N/A |

**References**

1. Abusalem SK, Coty M-B: **Home health nurses coping with practice care errors**. *Journal of Research in Nursing* 2013, **18**(4):336-348.

2. Ajoudani F, Habibzadeh H, Baghaei R: **Second Victim Experience and Support Tool: Persian translation and psychometric characteristics evaluation**. *International nursing review* 2020.

3. Ajri-Khameslou M, Abbaszadeh A, Borhani F: **Emergency Nurses as Second Victims of Error: A Qualitative Study**. *Advanced emergency nursing journal* 2017, **39**(1):68-76.

4. Bari A, Khan RA, Rathore AW: **Medical errors; causes, consequences, emotional response and resulting behavioral change**. *Pak J Med Sci* 2016, **32**(3):523-528.

5. Biggs S, Waggett HB, Shabbir J: **Impact of surgical complications on the operating surgeon**. *Colorectal Dis* 2020.

6. Brunelli MV, Estrada S, Celano C: **Cross-Cultural Adaptation and Psychometric Evaluation of a Second Victim Experience and Support Tool (SVEST)**. *J Patient Saf* 2018.

7. Burlison JD, Quillivan RR, Scott SD, Johnson S, Hoffman JM: **The Effects of the Second Victim Phenomenon on Work-Related Outcomes: Connecting Self-Reported Caregiver Distress to Turnover Intentions and Absenteeism**. *J Patient Saf* 2016.

8. Burlison JD, Scott SD, Browne EK, Thompson SG, Hoffman JM: **The Second Victim Experience and Support Tool: Validation of an Organizational Resource for Assessing Second Victim Effects and the Quality of Support Resources**. *Journal of patient safety* 2017, **13**(2):93-102.

9. Cebeci F, Karazeybek E, Sucu G, Kahveci R: **Nursing students’ medication errors and their opinions on the reasons of errors: A cross-sectional survey**. *Journal of the Pakistan Medical Association* 2015, **65**(5):457-462.

10. Chan ST, Khong BPC, Pei Lin Tan L, He HG, Wang W: **Experiences of Singapore nurses as second victims: A qualitative study**. *Nursing & health sciences* 2018, **20**(2):165-172.

11. Chen J, Yang Q, Zhao Q, Zheng S, Xiao M: **Psychometric validation of the Chinese version of the Second Victim Experience and Support Tool (C‐SVEST)**. *Journal of Nursing Management (John Wiley & Sons, Inc)* 2019, **27**(7):1416-1422.

12. Choi EY, Pyo J, Lee W, Jang SG, Park YK, Ock M, Lee SI: **Nurses' experiences of patient safety incidents in Korea: a cross-sectional study**. *BMJ Open* 2020, **10**(10):e037741.

13. Christoffersen L, Teigen J, Rønningstad C: **Following-up midwives after adverse incidents: How front-line management practices help second victims**. *Midwifery* 2020, **85**:102669.

14. Chung AS, Smart J, Zdradzinski M, Roth S, Gende A, Conroy K, Battaglioli N: **Educator Toolkits on Second Victim Syndrome, Mindfulness and Meditation, and Positive Psychology: The 2017 Resident Wellness Consensus Summit**. *Western Journal of Emergency Medicine: Integrating Emergency Care with Population Health* 2018, **19**(2):327-331.

15. Deringer E, Caligor E: **Supervision and responses of psychiatry residents to adverse patient events**. *Acad Psychiatry* 2014, **38**(6):761-767.

16. Dukhanin V, Edrees HH, Connors CA, Kang E, Norvell M, Wu AW: **Case: A Second Victim Support Program in Pediatrics: Successes and Challenges to Implementation**. *Journal of Pediatric Nursing* 2018, **41**:54-59.

17. Edrees HH, Wu AW: **Does One Size Fit All? Assessing the Need for Organizational Second Victim Support Programs**. *J Patient Saf* 2017.

18. Edrees HH, Morlock L, Wu AW: **Do Hospitals Support Second Victims? Collective Insights From Patient Safety Leaders in Maryland**. *Joint Commission Journal on Quality and Patient Safety* 2017, **43**(9):471-483.

19. Edrees HH, Paine LA, Feroli ER, Wu AW: **Health care workers as second victims of medical errors**. *Polskie Archiwum Medycyny Wewnetrznej* 2011, **121**(4):101-107.

20. Edrees H, Brock DM, Wu AW, McCotter PI, Hofeldt R, Shannon SE, Gallagher TH, White AA: **The experiences of risk managers in providing emotional support for health care workers after adverse events**. *Journal of Healthcare Risk Management* 2016, **35**(4):14-21.

21. Edrees H, Connors C, Paine L, Norvell M, Taylor H, Wu AW: **Implementing the RISE second victim support programme at the Johns Hopkins Hospital: A case study**. *BMJ Open* 2016, **6**(9).

22. El Hechi MW, Bohnen JD, Westfal M, Han K, Cauley C, Wright C, Schulz J, Mort E, Ferris T, Lillemoe KD *et al*: **Design and Impact of a Novel Surgery-Specific Second Victim Peer Support Program**. *Journal of the American College of Surgeons* 2020, **230**(6):926-933.

23. Finney RE, Torbenson VE, Riggan KA, Weaver AL, Long ME, Allyse MA, Rivera-Chiauzzi E: **Second Victim Experiences of Nurses in Obstetrics and Gynecology: a SVEST Survey**. *Journal of nursing management* 2020.

24. Graham P, Zerbi G, Norcross W, Montross-Thomas L, Lobbestael L, Davidson J: **Testing of A Caregiver Support Team**. *Explore* 2019, **15**(1):19-26.

25. Gupta K, Lisker S, Rivadeneira NA, Mangurian C, Linos E, Sarkar U: **Decisions and repercussions of second victim experiences for mothers in medicine (SAVE DR MoM)**. *BMJ Quality & Safety* 2019, **28**(7):564-573.

26. Habibzadeh H, Baghaei R, Ajoudani F: **Relationship between patient safety culture and job burnout in Iranian nurses: Assessing the mediating role of second victim experience using structural equation modelling**. *Journal of Nursing Management (John Wiley & Sons, Inc)* 2020, **28**(6):1410-1417.

27. Han K, Bohnen JD, Peponis T, Martinez M, Nandan A, Yeh DD, Lee J, Demoya M, Velmahos G, Kaafarani HMA: **The Surgeon as the Second Victim? Results of the Boston Intraoperative Adverse Events Surgeons’ Attitude (BISA) Study**. *Journal of the American College of Surgeons* 2017, **224**(6):1048-1056.

28. Harrison R, Lawton R, Stewart K: **Doctors' experiences of adverse events in secondary care: The professional and personal impact**. *Clinical Medicine, Journal of the Royal College of Physicians of London* 2014, **14**(6):585-590.

29. Harrison R, Lawton R, Perlo J, Gardner P, Armitage G, Shapiro J: **Emotion and coping in the aftermath of medical error: a cross-country exploration**. *Journal of patient safety* 2015, **11**(1):28-35.

30. Harrison R, Lee H, Sharma A: **A Survey of the Impact of Patient Adverse Events and near Misses on Anaesthetists in Australia and New Zealand**. *Anaesthesia and Intensive Care* 2018, **46**(5):510-515.

31. Harrison R, Sharma A, Walton M, Esguerra E, Onobrakpor S, Nghia BT, Chinh ND: **Responding to adverse patient safety events in Viet Nam**. *BMC health services research* 2019, **19**(1):677.

32. Huang H, Chen J, Xiao M, Cao S, Zhao Q: **Experiences and responses of nursing students as second victims of patient safety incidents in a clinical setting: A mixed‐methods study**. *Journal of Nursing Management (John Wiley & Sons, Inc)* 2020, **28**(6):1317-1325.

33. Joesten L, Cipparrone N, Okuno-Jones S, DuBose ER: **Assessing the Perceived Level of Institutional Support for the Second Victim After a Patient Safety Event**. *Journal of Patient Safety* 2015, **11**(2):73-78.

34. Kable A, Kelly B, Adams J: **Effects of adverse events in health care on acute care nurses in an Australian context: A qualitative study**. *Nursing & health sciences* 2018, **20**(2):238-246.

35. Kim EM, Kim SA, Lee JR, Burlison JD, Oh EG: **Psychometric Properties of Korean Version of the Second Victim Experience and Support Tool (K-SVEST)**. *Journal of patient safety* 2020, **16**(3):179-186.

36. Kobe C, Blouin S, Moltzan C, Koul R: **The Second Victim Phenomenon: Perspective of Canadian Radiation Therapists**. *Journal of Medical Imaging and Radiation Sciences* 2019, **50**(1):87-97.

37. Koehn AR, Ebright PR, Draucker CB: **Nurses' experiences with errors in nursing**. *Nursing outlook* 2016, **64**(6):566-574.

38. Krzan KD, Merandi J, Morvay S, Mirtallo J: **Implementation of a "second victim" program in a pediatric hospital**. *American Journal of Health-System Pharmacy* 2015, **72**(7):563-567.

39. Lane MA, Newman BM, Taylor MZ, OʼNeill M, Ghetti C, Woltman RM, Waterman AD: **Supporting Clinicians After Adverse Events: Development of a Clinician Peer Support Program**. *Journal of patient safety* 2018, **14**(3):e56-e60.

40. Luu S, Patel P, St-Martin L, Leung ASO, Regehr G, Murnaghan ML, Gallinger S, Moulton C-a: **Waking up the next morning: surgeons' emotional reactions to adverse events**. *Medical Education* 2012, **46**(12):1179-1188.

41. McCarthy SE, O'Boyle CA, O'Shaughnessy A, Walsh G: **Online patient safety education programme for junior doctors: is it worthwhile?** *Ir J Med Sci* 2016, **185**(1):51-58.

42. McLennan SR, Engel-Glatter S, Meyer AH, Schwappach DLB, Scheidegger DH, Elger BS: **The impact of medical errors on Swiss anaesthesiologists: A cross-sectional survey**. *Acta Anaesthesiologica Scandinavica* 2015, **59**(8):990-998.

43. Merandi J, Liao N, Lewe D, Morvay S, Stewart B, Catt C, Scott SD: **Deployment of a Second Victim Peer Support Program: A Replication Study**. *Pediatr Qual Saf* 2017, **2**(4):e031.

44. Merandi J, Winning AM, Liao N, Rogers E, Lewe D, Gerhardt CA: **Implementation of a second victim program in the neonatal intensive care unit: An interim analysis of employee satisfaction**. *Journal of Patient Safety & Risk Management* 2018, **23**(6):231-238.

45. Mira JJ, Carrillo I, Lorenzo S, Ferrús L, Silvestre C, Pérez-Pérez P, Olivera G, Iglesias F, Zavala E, Maderuelo-Fernández JA *et al*: **The aftermath of adverse events in Spanish primary care and hospital health professionals**. *BMC Health Services Research* 2015, **15**(1):151-151.

46. Mira JJ, Lorenzo S, Carrillo I, Ferrús L, Pérez-Pérez P, Iglesias F, Silvestre C, Olivera G, Zavala E, Nuño-Solinís R *et al*: **Interventions in health organisations to reduce the impact of adverse events in second and third victims**. *BMC Health Serv Res* 2015, **15**:341.

47. Mohamadi-Bolbanabad A, Moradi G, Piroozi B, Safari H, Asadi H, Nasseri K, Mohammadi H, Afkhamzadeh A: **The second victims’ experience and related factors among medical staff**. *International Journal of Workplace Health Management* 2019, **12**(3):134-145.

48. Mohsenpour M, Hosseini M, Abbaszadeh A, Shahboulaghi FM, Khankeh H: **Iranian nurses' experience of "being a wrongdoer": A phenomenological study**. *Nurs Ethics* 2018, **25**(5):653-664.

49. Mok WQ, Chin GF, Yap SF, Wang W: **A cross-sectional survey on nurses' second victim experience and quality of support resources in Singapore**. *J Nurs Manag* 2020, **28**(2):286-293.

50. Panella M, Rinaldi C, Leigheb F, Donnarumma C, Kul S, Vanhaecht K, Di Stanislao F: **The determinants of defensive medicine in Italian hospitals: The impact of being a second victim**. *Rev Calid Asist* 2016, **31 Suppl 2**:20-25.

51. Pijl Zieber M, Williams B: **The Experience of Nursing Students Who Make Mistakes in Clinical**. *International Journal of Nursing Education Scholarship* 2015, **12**(1):1-9.

52. Pinto A, Faiz O, Bicknell C, Vincent C: **Acute traumatic stress among surgeons after major surgical complications**. *American Journal of Surgery* 2014, **208**(4):642-647.

53. Pratt S, Kenney L, Scott SD, Wu AW: **How to develop a second victim support program: a toolkit for health care organizations**. *Joint Commission journal on quality and patient safety / Joint Commission Resources* 2012, **38**(5):235-240, 193.

54. Pyo J, Choi EY, Lee W, Jang SG, Park YK, Ock M, Lee SI: **Physicians' Difficulties Due to Patient Safety Incidents in Korea: a Cross-Sectional Study**. *J Korean Med Sci* 2020, **35**(17):e118.

55. Quillivan RR, Burlison JD, Browne EK, Scott SD, Hoffman JM: **Patient Safety Culture and the Second Victim Phenomenon: Connecting Culture to Staff Distress in Nurses**. *Joint Commission Journal on Quality & Patient Safety* 2016, **42**(8):377-AP372.

56. Reiser CF, Schwappach D, Schwendimann R: **Supporting health professionals after an adverse event in Swiss hospitals: A cross-sectional study**. *Swiss Medical Weekly* 2020, **150**(25-26).

57. Rinaldi C, Leigheb F, Vanhaecht K, Donnarumma C, Panella M: **Becoming a "second victim" in health care: Pathway of recovery after adverse event**. *Rev Calid Asist* 2016, **31 Suppl 2**:11-19.

58. Scheepstra KWF, Pauw HS, van Steijn ME, Stramrood CAI, Olff M, van Pampus MG: **Potential traumatic events in the workplace and depression, anxiety and post-traumatic stress: a cross-sectional study among Dutch gynaecologists, paediatricians and orthopaedic surgeons**. *BMJ Open* 2020, **10**(9):e033816.

59. Schrøder K, Edrees HH, Christensen RD, Jørgensen JS, Lamont RF, Hvidt NC: **Second victims in the labor ward: Are Danish midwives and obstetricians getting the support they need?** *International Journal for Quality in Health Care* 2019, **31**(8):583-589.

60. Stangierski A, Warmuz-Stangierska I, Ruchala M, Zdanowska J, Glowacka MD, Sowinski J, Ruchala P, Ruchała M, Głowacka MD, Sowiński J *et al*: **Medical errors - not only patients' problem**. *Archives of Medical Science* 2012, **8**(3):569-574.

61. Stone M: **Clinical nurses' experiences with sentinel events**. *Nursing Management* 2019, **50**(11):38-42.

62. Stone M: **Second Victim Support: Nurses' Perspectives of Organizational Support After an Adverse Event**. *The Journal of nursing administration* 2020, **50**(10):521-525.

63. Strametz R, Siebold B, Heistermann P, Haller S, Bushuven S: **Validation of the German Version of the Second Victim Experience and Support Tool-Revised**. *J Patient Saf* 2021.

64. Strametz R, Koch P, Vogelgesang A, Burbridge A, Rösner H, Abloescher M, Huf W, Ettl B, Raspe M: **Prevalence of second victims, risk factors and support strategies among young German physicians in internal medicine (SeViD-I survey)**. *J Occup Med Toxicol* 2021, **16**(1):11-11.

65. Stukalin I, Lethebe BC, Temple W: **The physician's Achilles heel-surviving an adverse event**. *Curr Oncol* 2019, **26**(6):e742-e747.

66. Swartwout E, Rodan M: **The Development and Testing of the Psychometric Properties of the Emotional Response and Disclosure of Errors in Clinical Practice Instrument**. *Journal of Nursing Measurement* 2017, **25**(1):184-200.

67. Tan R, Luo K, Hu D, Zhao Y, Han Y, Xu K: **Inpatient Suicide Second Victim Experience and Support Tool: Psychometric properties of a scale for nurses who experienced inpatient suicide at Chinese general hospitals**. *Nurs Health Sci* 2020.

68. Treiber LA, Jones JH: **Making an Infusion Error: The Second Victims of Infusion Therapy-Related Medication Errors**. *Journal of Infusion Nursing* 2018, **41**(3):156-163.

69. Tumelty ME: **The Second Victim: A Contested Term?** *J Patient Saf* 2018.

70. Ullström S, Sachs MA, Hansson J, Øvretveit J, Brommels M: **Suffering in silence: A qualitative study of second victims of adverse events**. *BMJ Quality and Safety* 2014, **23**(4):325-331.

71. Van Gerven E, Bruyneel L, Panella M, Euwema M, Sermeus W, Vanhaecht K: **Psychological impact and recovery after involvement in a patient safety incident: A repeated measures analysis**. *BMJ Open* 2016, **6**(8).

72. Van Gerven E, Deweer D, Scott SD, Panella M, Euwema M, Sermeus W, Vanhaecht K: **Personal, situational and organizational aspects that influence the impact of patient safety incidents: A qualitative study**. *Revista de calidad asistencial : organo de la Sociedad Espanola de Calidad Asistencial* 2016, **31**:34-46.

73. Van Gerven E, Elst TV, Vandenbroeck S, Dierickx S, Euwema M, Sermeus W, De Witte H, Godderis L, Vanhaecht K, Vander Elst T: **Increased Risk of Burnout for Physicians and Nurses Involved in a Patient Safety Incident**. *Medical Care* 2016, **54**(10):937-943.

74. Vanhaecht K, Seys D, Schouten L, Bruyneel L, Coeckelberghs E, Panella M, Zeeman G: **Duration of second victim symptoms in the aftermath of a patient safety incident and association with the level of patient harm: a cross-sectional study in the Netherlands**. *BMJ Open* 2019, **9**(7):e029923.

75. Vinson AE, Mitchell JD: **Assessing levels of support for residents following adverse outcomes: a national survey of anesthesia residency programs in the United States**. *Medical teacher* 2014, **36**(10):858-866.

76. Wahlberg Å, Högberg U, Emmelin M: **Left alone with the emotional surge - A qualitative study of midwives' and obstetricians' experiences of severe events on the labour ward**. *Sex Reprod Healthc* 2020, **23**:100483.

77. White AA, Brock DM, McCotter PI, Hofeldt R, Edrees HH, Wu AW, Shannon S, Gallagher TH: **Risk managers' descriptions of programs to support second victims after adverse events**. *Journal of healthcare risk management : the journal of the American Society for Healthcare Risk Management* 2015, **34**(4):30-40.

78. Winning AM, Merandi JM, Lewe D, Stepney LMC, Liao NN, Fortney CA, Gerhardt CA: **The emotional impact of errors or adverse events on healthcare providers in the NICU: The protective role of coworker support**. *Journal of advanced nursing* 2018, **74**(1):172-180.

79. Winning AM, Merandi J, Rausch JR, Liao N, Hoffman JM, Burlison JD, Gerhardt CA: **Validation of the Second Victim Experience and Support Tool-Revised in the Neonatal Intensive Care Unit**. *Journal of patient safety* 2020.

80. Yung HP, Yu S, Chu C, Hou IC, Tang FI: **Nurses' attitudes and perceived barriers to the reporting of medication administration errors**. *Journal of Nursing Management (John Wiley & Sons, Inc)* 2016, **24**(5):580-588.

81. Zeeman G, Schouten L, Seys D, Coeckelberghs E, Weijenborg P, Bruyneel L, Vanhaecht K: **Prolonged mental health sequelae among doctors and nurses involved in patient safety incidents with formal complaints and lawsuits**. *Eur J Public Health* 2020, **30**(4):777-779.

82. Zhang X, Chen J, Lee SY: **Psychometric Testing of the Chinese Version of Second Victim Experience and Support Tool**. *J Patient Saf* 2020.

83. Zhang X, Li Q, Guo Y, Lee SY: **From organisational support to second victim‐related distress: Role of patient safety culture**. *Journal of Nursing Management (John Wiley & Sons, Inc)* 2019, **27**(8):1818-1825.

84. Scott SD: **The second victim experience: Mitigating the harm**. *American Nurse Today* 2015, **10**(9):8-11.

85. Schrøder K, Hvidt NC, Jørgensen JS, Lamont RF, Jørgensen JS, Lamont RF, Hvidt NC: **Second victims need emotional support after adverse events: even in a just safety culture**. *BJOG: An International Journal of Obstetrics & Gynaecology* 2019, **126**(4):440-442.

86. Tebala GD: **Is there a standard reaction of surgeons to surgical complications? Study on an interesting historical case**. *Med Hypotheses* 2020, **144**:110006.

87. Sataloff RT: **Adverse Surgical Events: Effects on the Surgeon**. *Ear, Nose and Throat Journal* 2020, **99**(4):225-226.

88. MacLeod L: **"Second victim" casualties and how physician leaders can help**. *Physician executive* 2014, **40**(1):8-12.

89. Rappaport DI, Selbst SM, Mull CC: **Medical Errors and Malpractice Lawsuits: Impact on Providers- Part 2 of 6**. *Pediatric Emergency Care* 2019, **35**(6):440-442.

90. Thompson CV, Suggett N, Fellows J: **Must "second victims" always be in the wrong?** *BMJ (Online)* 2015, **350**.

91. Anderson J, Gianola FJ: **After the error, then what? The emotional impact of errors on clinicians**. *JAAPA: Journal of the American Academy of Physician Assistants (Haymarket Media, Inc)* 2011, **24**(12):71-72.

92. Everly Jr GS: **Psychological first aid to support healthcare professionals**. *Journal of Patient Safety & Risk Management* 2020, **25**(4):159-162.

93. Shapiro J, Galowitz P: **Peer Support for Clinicians: A Programmatic Approach**. *Academic medicine : journal of the Association of American Medical Colleges* 2016, **91**(9):1200-1204.

94. Hauk L: **Support strategies for health care professionals who are second victims**. *AORN journal* 2018, **107**(6):P7-P9.

95. Edrees H, Federico F: **Supporting clinicians after medical error**. *BMJ (Online)* 2015, **350**.

96. Smetzer J: **Don't abandon the "second victims" of medical errors**. *Nursing* 2012, **42**(2):54-58.

97. Saver C: **'Second victim' rapid-response team helps fellow clinicians recover from trauma**. *OR manager* 2013, **29**(5):10-12, 27.

98. Scott SD, McCoig MM: **Care at the point of impact: Insights into the second-victim experience**. *Journal of healthcare risk management : the journal of the American Society for Healthcare Risk Management* 2016, **35**(4):6-13.

99. [<http://m.hopkinsmedicine.org/news/publications/dome/november_2010/the_second_victims>]

100. Slykerman G, Wiemers MJ, Wyssusek KH: **Peer support in anaesthesia: Development and implementation of a peer-support programme within the Royal Brisbane and Women’s Hospital Department of Anaesthesia and Perioperative Medicine**. *Anaesthesia and Intensive Care* 2019, **47**(6):497-502.

101. [<http://www.brighamandwomens.org/medical_professionals/career/cpps/PeerSupport.aspx>]

102. [<http://mikegreenstein.com/media/docs/Physicians-Insurance-Annual-Report-2010.pdf>]

103. [<https://www.phyins.com/magazine/provider-support-program>]

104. [<http://www.ihi.org/resources/Pages/Publications/SupportingInvolvedHealthCareProfessionalsSecondVictims.aspx> ]

105. [<https://www.uclahealth.org/quality/Workfiles/quality/NQF-Safety.pdf>]

106. [<https://www.theschwartzcenter.org/about/who-we-are/>]

107. [<http://www.mitss.org/>]

108. [<https://www.ismp-canada.org/download/safetyBulletins/2017/ISMPCSB2017-10-SecondVictim.pdf>]

109. [<https://www.muhealth.org/sites/default/files/forYOUstaff_brochure.pdf>]

110. [<https://healthynh.org/images/Implementationofasecondvictimprograminapediatrichospital_2.pdf>]

111. [<http://kpnet.kp.org/qrrm/risk/CareforCaregivers/care_for_caregivers.htm>]

112. Denham CR: **TRUST: the 5 rights of the second victim**. *Journal of Patient Safety* 2007, **3**(2):107-119.

113. [<https://secondvictim.be/richtlijn-in-dutch-only/>]

114. [<https://www.uic.edu/apps/departments-az/search?dispatch=find&orgid=94765>]

115. [<https://www.aana.com/docs/default-source/aana-journal-web-documents-1/design-second-victim-0416-pp107-113.pdf?sfvrsn=f1d448b1_6>]

116. [<https://www.patientensicherheit.ch/>]

117. [<https://digitalcommons.gardner-webb.edu/cgi/viewcontent.cgi?article=1025&context=nursing_etd>]

118. [<https://www.anzca.edu.au/about-us/doctors-health-and-wellbeing>]

119. [<https://rcoa.ac.uk/sites/default/files/documents/2019-11/Guideline_catastrophes_anaesthetic_practice_2005_final.pdf>]

120. Mitchell I, Schuster A, Smith K, Pronovost P, Wu A: **Patient safety incident reporting: a qualitative study of thoughts and perceptions of experts 15 years after ‘<em>To Err is Human</em>’**. *BMJ Quality &amp; Safety* 2016, **25**(2):92-99.

121. [<http://www.segundasvictimas.es/acceso.php>]

122. [<https://www.icisf.org/>]
